# The relationship between healthcare access and change in health-related quality-of-life among the general population of five countries during the COVID-19 pandemic

**DOI:** 10.1007/s11136-024-03704-1

**Published:** 2024-06-11

**Authors:** Nadja Alexandrov, Emily Stella Scott, Mathieu F. Janssen, Erica I. Lubetkin, John N. Yfantopoulos, Gouke J. Bonsel, Juanita A. Haagsma

**Affiliations:** 1 Department of Public Health, Erasmus MC, Rotterdam, The Netherlands; 2 Section Medical Psychology and Psychotherapy, Department of Psychiatry, Erasmus MC, Rotterdam, The Netherlands; 3 Department of Community Health and Social Medicine, CUNY School of Medicine, New York City, NY USA; 4 Health Department of Economics, National and Kapodistrian University of Athens, Athens, Greece; 5 Department Scientific Support, EuroQol Research Foundation, Rotterdam, The Netherlands

**Keywords:** Quality of life, Epidemiology, Chronic disease, Population health, Healthcare disparities, COVID-19

## Abstract

**Purpose:**

To determine whether (1) healthcare access at onset of the pandemic and (2) age, gender, socioeconomic status (SES), and pre-existing health status were associated with change in health-related quality-of-life (HRQoL) during the COVID-19 pandemic. The study includes a general population sample of five countries.

**Methods:**

An online questionnaire was administered to respondents from Greece, Italy, the Netherlands, the UK, and the US at the onset of the COVID-19 pandemic between April 22nd and May 5th of 2020, and 1 year later between May 23rd and June 29th of 2021. The questionnaire included questions on demographic background, health status, and HRQoL. The primary outcome was change in HRQoL as measured by the EQ-5D-5L instrument. Specifically, the EQ-5D-5L index and EQ VAS were used. Healthcare access was quantified with regard to the respondent’s ease of getting an appointment, waiting time, and opportunity to contact the provider and during analysis dichotomized into “sufficient” versus “insufficient”. Linear regression analysis was performed with change in HRQoL as dependent variable and background variables as independent variables.

**Results:**

In total, 6,765 respondents completed the second questionnaire. 19.8% of total respondents reported insufficient healthcare access. Respondents with insufficient healthcare had both more improved and deteriorated HRQoL compared to respondents with sufficient healthcare, whose HRQoL remained unchanged. We did not find significant interactions between age, gender, SES and/or chronic disease status with healthcare access at onset of the COVID-19 pandemic.

**Conclusion:**

Healthcare access was not associated with cumulative differences in change in HRQoL over a 1-year period in strata of age, gender, SES, and chronic disease status.

**Supplementary Information:**

The online version contains supplementary material available at 10.1007/s11136-024-03704-1.

## Plain english summary

The coronavirus (COVID-19) pandemic caused unprecedented challenges across healthcare systems worldwide due to the high number of COVID-19 infections. As COVID-19 infections had to be prioritised, surgeries had to be postponed and routine appointments were harder to get by. This resulted in a substantial decline in access to healthcare services. In addition, the COVID-19 pandemic disproportionately affected those with disadvantaged backgrounds, such as those having a lower education or with pre-existing poor health conditions. It is important to understand how sub-optimal access to healthcare can impact an individual’s overall health and well-being, and whether it impacts more disadvantaged populations differently. Health-Related Quality-of-Life (HRQoL) is a good summary measure of an individual’s overall health, as it summarises their level of perception of their physical, psychological and social aspects of life.

This study is concerned with understanding whether non-optimal access to healthcare during the COVID-19 pandemic could have impaired HRQoL over time of the general population. More specifically, we are interested in understanding whether levels of HRQoL differed in people with more disadvantaged backgrounds.

The results showed that individuals in this study with disadvantaged backgrounds and pre-existing health conditions did not have differing levels of HRQoL due to a change in healthcare access. This demonstrated a negligible impact of healthcare access on the change in HRQoL.

## Introduction

### Background

The COVID-19 pandemic has significantly impacted population health worldwide [1]. Since the start of the pandemic in 2020 more than 797 million confirmed cases and 6.9 million deaths due to COVID-19 infection have been reported [2]. The effects of the COVID-19 pandemic are not limited to consequences of infection, but also include downstream and long-term effects on the global economy, at the healthcare level, and in the daily lives of individuals [3]. Such effects encompass both economic and social effects due to direct effects of COVID-19, such as acute COVID-19 infection and post COVID-19 condition, as well as indirect effects, such as government-mandated lockdowns and quarantines.

Of special concern has been the limited access to healthcare for COVID-19 patients and non-COVID patients during the pandemic phase [4]. Healthcare workers were affected both physically (e.g. by exhaustion and COVID-19 infection) and psychologically (e.g. by self-reported stress and anxiety) [5–8]. The impact of the workload, risks and psychological burden translated to decreased working capacity [5, 9]. Additionally, the waxing and waning of the number of COVID-19 cases during the pandemic interfered with capacity-planning.

During the COVID-19 pandemic, the main priority was treatment of acute COVID-19 infection cases and regular care was scaled down or postponed [10–14]. Consequently, individuals living with chronic disease during the COVID-19 pandemic experienced reduced access to healthcare services: especially with regard to in-person visits and medication supply [12, 15, 16]. Apart from limited healthcare access, chronic disease patients faced increased risk of severe complications and mortality upon COVID-19 contraction [17]. Taken together, chronic disease caused considerable distress during the COVID-19 pandemic, adding to the already existent health-related quality of life (HRQoL) impairments for these individuals [18].

The impact of the COVID-19 pandemic on HRQoL reportedly is substantial and varies with demographic, socio-economic, as well as health-related factors for both patients and wider populations [19]. The pandemic has exposed existing health inequalities through at least two different pathways: having a disadvantaged background limits healthcare access in general, and a disadvantaged background increases chronic disease prevalence and worsens prognosis, with the described HRQoL impact [3, 13, 20]. Accumulation of negative COVID-19 effects in the general population has indeed been demonstrated in the US (New York) among those with a lower obtained level of education and those with poorer healthcare access [3], in Germany among children and adolescents with socially disadvantaged backgrounds and low socioeconomic status (SES) [21], and among Polish adults with low education attainment [22]. The pathway through chronic disease was observed in China and Ethiopia [23, 24].

As stated, limited access to healthcare, and the fear of contracting COVID-19 in those with chronic disease may have resulted in a cumulative deterioration of HRQoL [20, 25], regardless of country of origin. For instance, it is reported that individuals with rare diseases in the US have low HRQoL, which is related to barriers in healthcare access in general, and patients with chronic disease in Ethiopia had low HRQoL during the COVID-19 pandemic, where missed healthcare visits were significantly associated with impaired HRQoL [24, 25].

It is important to understand differences in HRQoL associated with healthcare access for respondents with disadvantaged backgrounds, in order to address unmet healthcare needs in these individuals. Our study adds to current knowledge by revealing the cumulative association between healthcare access and HRQoL for people from marginalized backgrounds during the COVID-19 pandemic, leveraging a multi-country design. It examines the general role of age, and gender, the specific role of socioeconomic status (i.e. educational attainment) and pre-existing health status (i.e. chronic disease presence), as well as the role of limited healthcare access. The timing of the data collection allowed us to include the early phase of the pandemic, during its first peak in early 2020, while the repeated data collection approximately 1 year later (2021) permitted a longitudinal analysis.

### Objectives

To determine to what extent (1) healthcare access at onset of the pandemic, (2) sociodemographic background (age, gender, SES), and pre-existing health status predict deteriorated HRQoL during the COVID-19 pandemic. The study includes a general population sample of five countries.

### Research questions

1. To what extent does insufficient healthcare access influence HRQoL during the COVID-19 pandemic over a 1-year period (T2-T1) in the general population stratified by age, gender, SES, and disease status (no chronic disease or one or more chronic disease(s))?
2. Does age, gender, SES, and/or chronic disease act as moderators of healthcare access in the relationship between healthcare access at T1 and change in HRQoL?

**Fig. 1:**
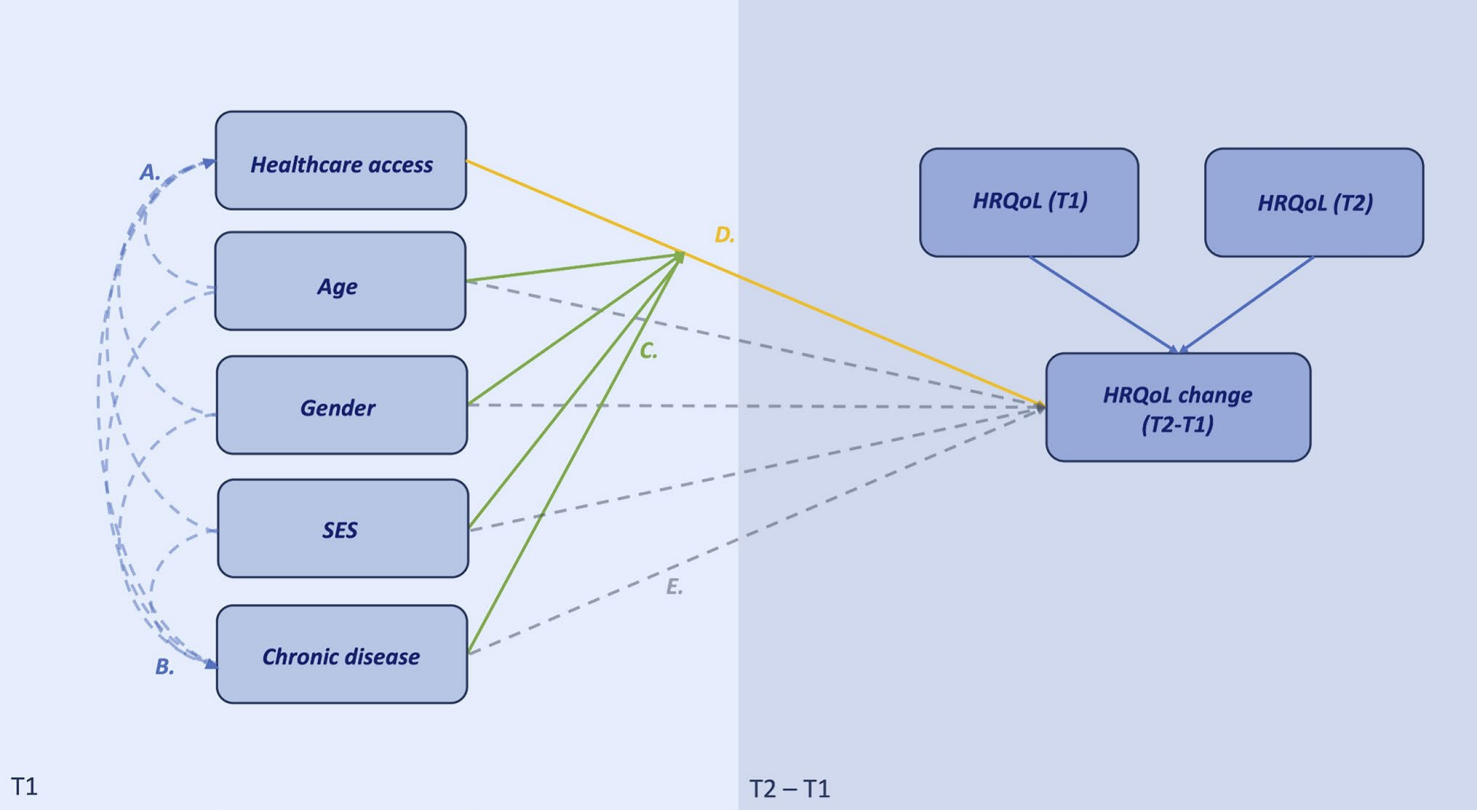
Conceptual model of hypothesized relationships concerning healthcare access and HRQoL including controlled background variables (age, gender, SES, chronic disease status). Sociodemographic factors (age, gender, SES) may act directly on healthcare access (pathway A), on chronic disease prevalence and subsequently severity (pathway B), as moderators of the relationship between healthcare access and HRQoL change (pathway C), and directly on HRQoL change (pathway E). Insufficient healthcare access may directly impact HRQoL change (D). We are exploring an interaction effect between (1) age, gender, SES, and chronic disease, and HRQoL change via healthcare access, and (2) the direct association of these variables with HRQoL change, controlling for healthcare access

Access to healthcare at T1 is hypothesized to have a cumulative effect on change in HRQoL, with the smallest effect in the least deprived stratum (male, 18 to 34 years old, high SES, with no chronic disease) and largest effect in the most deprived stratum (female, 55 to 75 years old, low SES, at least one chronic disease). For conceptual model, see  Fig. 1

## Methods

### Study design and data collection

This study is part of the POPulation health impact of the CORoNavirus (COVID-19) study (POPCORN), which is a longitudinal study that measures the impact of the COVID-19 pandemic on the HRQoL and mental well-being of the general population in Greece, Italy, the Netherlands, the United Kingdom (UK), and the United States (US). In the POPCORN study, participants from the general population agreed to participate in a survey that was administered through an online platform by an international market research agency. The first wave of data collection was obtained between April 22nd and May 5th of 2020 and the second wave was between May 23rd and June 29th of 2021. Data obtained were anonymized, and ethical approval was obtained from the Erasmus MC ethics review board (approval MEC-2020-0266). The participant recruitment and questionnaire distribution were performed by Dynata. Existing large internet panels from all countries were used, and these samples were designed to be representative of the population aged 18–75 years in each country with regard to age, sex, and educational level, thus we employed a quota-sampling strategy. Respondents were registered as members of the market research agency’s existing voluntary panels. Dropout rates (respondents who started the questionnaire but did not finish) can be found in Tables S1a and b (supplementary materials). Written informed consent was obtained upon registration as panel member. Once the questionnaire was started, the data capture system did not allow for missing values. Participants received an incentive in the form of cash or points from the research agency upon completing the questionnaire. Additional details of the data collection and response are available [1].

### Respondent characteristics at T1

Health-related determinants were assessed in the survey, including COVID-19 infection status and chronic disease status (“no chronic disease” or “one or more chronic disease(s)”). Sociodemographic determinants included gender (male or female), age at T1, highest achieved education level (categorized according to the International Standard of Classification of Education (ISCED) into low, medium, or high [26], occupation status (student, employed, unemployed, retired, or unable to work), living situation (living with others, living alone, or other), and healthcare access (sufficient or insufficient). Additional information on response categories and categorization of independent variables can be found in Table S2. Outpatient healthcare access was measured with the question: “thinking of your last visit, what was your experience with regard to access?”. “Access” was considered with regard to the respondent’s ease of getting an appointment, waiting time, and opportunity to contact the provider. There were two versions of responses, which ranged from “always good” to “never good” or “very good” to “very bad” on a five-point scale. The variable “outpatient care access” was conveniently dichotomized into “sufficient” versus “insufficient”. Sufficient access comprises the response levels “always good” and “usually good” and “very good” and “good”, whereas insufficient access comprises the remaining three response levels “sometimes good”, “usually not good”, and “never good”, and likewise “fair”, “bad”, and “very bad” were combined. Means and SD are reported for normally-distributed variables, whereas medians and IQR were reported for non-normally distributed variables.

### Primary outcome measures

The primary outcome measure in this study was *change in HRQoL* (T1 subtracted from T2) over a 1-year period, measured by the EQ-5D-5L instrument. The EQ-5D-5L is a measure of HRQoL that comprises five dimensions of health: mobility, self-care, usual activities, pain/discomfort, and anxiety/depression with a reference period of “your health today” [27]. Dimension responses can be aggregated unweighted or summated through specific weighting coefficients (value set) into an index score. The EQ-5D-5L index is a weighted summary score reflecting how a particular health state is valued by a representative sample of the general population from a specific region or country. In this study, the US value set was used to calculate the EQ-5D-5L index with scores ranging from −0.573 (worst possible score) to 1 (best possible score) [28]. For multi-country studies, the use of a single value set is suggested, in order to facilitate comparisons across countries. This allows us to compare differences in HRQoL rather than differences in HRQoL and differences in value set [29]. The EQ instrument also contains a visual analog scale (EQ VAS) where individuals rate their health on a scale from 0 to 100 [28]. The EQ VAS anchors are 'worst imaginable health', which refers to states worse than death (0), and 'best imaginable health’ (100).

### Statistical analyses

Change in HRQoL was calculated by subtracting the EQ-5D-5L scores at T1 from T2 for the EQ-5D-5L index and EQ VAS. Descriptive analyses were then performed on HRQoL (cross-sectional) and HRQoL changes using sociodemographic data, and health-related data as determinants. A positive difference score (EQ-5D-5L index, EQ VAS) denotes improved HRQoL. Positive regression coefficients for change in EQ-5D-5L index and EQ VAS scores thus imply a positive contribution to HRQoL improvement. Paired-Samples T Tests were conducted to investigate magnitude and direction of change of HRQoL between T1 and T2. Predictor variables were: COVID-19 status at T1, age category, living situation, gender, healthcare access, education level, and occupation.

Thirty-six multi-stratified regressions were run for both the EQ-5D-5L index and EQ VAS. For each individual regression, a unique combination of age-gender-SES-chronic disease constituted one stratum. Regressions were univariate with dichotomized independent variable “outpatient care access” and continuous dependent variables EQ-5D-5L index change (T2-T1) and EQ VAS change (T2-T1). Regression coefficients were reported in 6 by 6 tables. Lastly, a full factorial univariate general linear model was performed. Based on the results, we built two models with change in HRQoL (EQ-5D-5L index and EQ VAS) as outcome variables. The significance level was set to 5%. All statistical analyses were carried out using IBM SPSS Statistics version 28.0.1.0. Sankey diagrams were created using RStudio using package networkD3. Bar charts were created with Windows Office Excel 2016.

## Results

### Study population

In total, 6,765 respondents completed the questionnaire at T2. The response rate was 41% at T2 (16,683 people responded at T1). Results of non-response bias analysis have been published previously [19]. Respondents at T2 were significantly older than those who did not respond at T2, had less chronic conditions, and had different education levels and occupations. Table 1 and S2 show baseline characteristics of respondents at T1. Out of all respondents at T2, 511 were from Greece, 1,784 from Italy, 1,143 from the Netherlands, 1,448 from the UK, and 1,879 from the US. Most respondents were between 55 and 75 years old (43.6%). The age category of 18 to 34 years old contained the lowest number of respondents compared to any other age category: 1,013 respondents, 15% of the total respondents. The vast majority of respondents were highly educated (55.2%) and employed (53.5%). Furthermore, there were more female (52.3%) than male (47.7%) respondents. The Netherlands had a large proportion of low-educated respondents (29%) compared to the total of all countries (10.3%) (Table S3). Moreover, 3,888 (57.5%) respondents had no chronic disease, somewhat more than with at least one chronic disease (42.5%).

**Table 1:** Baseline characteristics for all respondents at T1, total and by outpatient care access (sufficient versus insufficient)

			Outpatient care access	
Response rate (T2/T1)			6765 (41%)	
		Total	Sufficient	Insufficient
Number of respondents		6765 (100%)	5427 (80.2%)	1338 (19.8%)
Age category				
	Median (IQR)	51.00 (23.00)	53.0 (23.0)	46.0 (21.0)
	Mean (SD)	50.84 (14.08)	51.9 (14.0)	46.8 (13.5)
	18–34	1013 (15.0%)	722 (71.3%)	291 (28.7%)
	35–54	2805 (41.5%)	2179 (77.7%)	626 (22.3%)
	55–75	2947 (43.6%)	2526 (85.7%)	421 (14.3%)
Gender	Male	3226 (47.7%)	2600 (80.6%)	626 (19.4%)
	Female	3539 (52.3%)	2827 (79.9%)	712 (20.1%)
Living situation	Living with others	5344 (79.0%)	4,305 (80.6%)	1039 (19.4%)
	Living alone	1288 (19.0%)	1025 (79.6	263 (20.4%)
	Other	133 (2.0%)	97 (72.9%)	36 (27.1%)
Education	High	3733 (55.2%)	3004 (80.5%)	729 (19.5%)
	Middle	2332 (34.5%)	1861 (79.8%)	471 (20.2%)
	Low	700 (10.3%)	562 (80.3%)	138 (19.7%)
Chronic disease status	No chronic disease	3888 (57.5%)	3176 (81.7%)	712 (18.3%)
	One or more chronic disease(s)	2877 (42.5%)	2251 (78.2%)	626 (21.8%)
COVID-19 infection status	Not infected	6622 (97.9%)	5330 (80.5%)	1292 (19.5%)
	Infected	143 (2.1%)	97 (67.8%)	46 (32.2%)
Occupation status	Employed	3622 (53.5%)	2881 (79.5%)	741 (20.5%)
	Student	174 (2.6%)	132 (75.9%)	42 (24.1%)
	Unemployed	1067 (15.8%)	784 (73.5%)	283 (26.5%)
	Retired	1550 (22.9%)	1369 (88.3%)	181 (11.7%)
	Unable to work	352 (5.2%)	261 (74.1%)	91 (25.9%)
Country	Greece	511 (7.6%)	362 (70.8%)	149 (29.2%)
	Italy	1784 (26.4%)	1353 (75.8%)	431 (24.2%)
	Netherlands	1143 (16.9%)	988 (86.4%)	155 (13.6%)
	UK	1448 (21.4%)	1064 (73.5%)	384 (26.5%)
	US	1879 (27.8%)	1660 (88.3%)	219 (11.7%)

### Insufficient healthcare access

Insufficient healthcare access was reported by 19.8% of respondents (Table 1) and ranged from 11.7% in the US (lowest) to 29.2% in Greece (highest), also reported in Table S3. Outpatient care access by age category by country is reported in Table S4. Table 1 with percentages shown by column can be found in Table S5. Total median age differed significantly between those who experienced insufficient outpatient care access and those with sufficient outpatient care access: 46.0 (IQR 21.0) and 53.0 (IQR 23.0) respectively (Table 1). Compared to insufficient healthcare access, a higher percentage of sufficient outpatient care access was observed across every age category. Overall, outpatient care access varied significantly across age categories. The distribution of age categories was significantly different between sufficient and insufficient outpatient care access (p < 0.001). 18 to 34-year-olds reported insufficient outpatient care access most out of all age categories (28.7%) and 55 to 75-year-olds the least (14.3%). Within the insufficient access category, most respondents were from Italy (32.2%), did not have chronic disease (53.2%), did not report having a COVID-19 infection at T1 (96.6%), and reported being employed (55.4%) (Table S5). Additionally, insufficient healthcare access was significantly higher among respondents with at least one chronic disease (21.8%) compared to those with no chronic disease (18.3%), presence of COVID-19 infection (32.2%) compared to those with no COVID-19 infection (19.5%), occupational status “unemployed” (26.5%) compared to other occupation categories, and being a respondent from Greece (29.2%) compared to other countries (Table 1).

### Effect of age, gender, SES, and chronic disease status on healthcare access and HRQoL change

Flow of change in EQ-5D-5L index and EQ-VAS stratified by healthcare access is shown in Figure S1. Change in HRQoL when accounting for healthcare access alone is shown in Table S6. Tables 2 and 3 show the results of the univariate linear regression analyses of EQ-5D-5L index change and EQ VAS change respectively. Number of respondents per stratum can be found in Table S7. Sub-group analyses were conducted using a subset of variables for stratification, as well as respondents’ last outpatient care visit, and can be found in Tables S8 through S14 and Figures S2 and S3. Overall, healthcare access was not significantly associated with HRQoL change when stratified by age, gender, SES, and chronic disease status for most strata. We can elucidate interaction effects of age, gender, SES, and chronic disease and healthcare access on HRQoL change by comparing equals, e.g. one can compare interaction effects with chronic disease status in the first cell in Table 3: Δ0.771 (confidence interval (CI): −5.202, 6.745) with the fourth cell Δ5.062 (CI −4.357, 14.481).

**Table 2:** Univariate linear regression analysis of sufficient compared to insufficient healthcare access on EQ-5D-5L index change (T2-T1)

		Chronic disease status					
		No chronic disease			One or more chronic disease(s)		
		SES			SES		
Gender	Age	High	Middle	Low	High	Middle	Low
Male	18–34	−0.055 (−0.117–0.007)	−0.047 (−0.138–0.044)	−0.002 (−0.207–0.202)	0.025 (−0.117–0.168)	0.036 (−0.066–0.139)	−0.062 (−0.452–0.328)
	35–54	−0.023 (−0.056–0.011)	0.037* (0.001–0.073)	0.017 (−0.119–0.153)	−0.062* (−0.166–−0.007)	−0.008 (−0.060–0.043)	−0.075 (−0.184–0.035)
	55–75	0.022 (−0.002–0.047)	0.009 (−0.019–0.037)	−0.014 (−0.066–0.028)	−0.017 (−0.050–0.015)	0.013 (−0.040–0.065)	−0.009 (−0.084–0.066)
Female	18–34	−0.005 (−0.045–0.036)	−0.019 (−0.097–0.058)	0.061 (−0.117–0.239)	0.002 (-−0.074–0.079)	−0.043 (−0.147–0.061)	0.012 (−0.285–0.309)
	35–54	−0.009 (−0.038–0.019)	−0.003 (−0.045–0.039)	−0.029 (−0.112–0.054)	−0.025 (−0.068–0.017)	0.000 (−0.056–0.056)	0.041 (−0.077–0.159)
	55–75	−0.031(−0.064–0.001)	0.027 (−0.007–0.061)	−0.025 (−0.075–0.025)	0.001 (−0.044–0.046)	−0.039 (−0.089–0.010)	−0.058 (−0.131–0.016)

Regression coefficients of 36 univariate regressions to determine the relationship of outpatient care access (sufficient vs insufficient) on EQ-5D-5L index change (T2-T1) using different strata (age, gender, SES, chronic disease status). Asterisks and bold text denote significant results (p < 0.05). 95% Confidence intervals are shown in parentheses

**Table 3:** Univariate linear regression analysis of sufficient compared to insufficient healthcare access on EQ VAS change (T2-T1)

		Chronic disease status					
		No chronic disease			One or more chronic disease(s)		
		SES			SES		
Gender	Age	High	Middle	Low	High	Middle	Low
Male	18–34	0.771 (−5.202–6.745)	−4.288 (−11.359–2.782)	0.000 (−23.718–23.718)	5.062 (−4.357–14.481)	5.071 (−5.440–15.583)	26.250 (−170.453–222.953)
	35–54	−0.064 (−2.914–2.785)	1.571 (−2.406–5.547)	−1.893 (−8.654–4.869)	−3.495 (−7.588–0.598)	−3.329 (−8.013–1.355)	−0.792 (−11.655–10.072)
	55–75	0.891(−2.410–4.191)	3.688* (0.474–6.901)	−7.009* (−12.427–−1.590)	−2.105 (−5.650–1.440)	−1.266 (−6.253–3.721)	0.282 (−9.235–9.800)
Female	18–34	−0.391 (−4.645–3.863)	4.056 (−3.115–11.227)	−2.000 (−16.638–12.638)	−1.362 (−7.525–4.800)	0.192 (−8.889–9.273)	−0.667 (−13.608–12.275)
	35–54	−0.357 (−3.419–2.705)	3.538 (−1.153–8.228)	2.326 (−6.462–11.214)	−0.241 (−4.834–4.351)	2.507 (−3.323–8.337)	−4.158 (−15.363–7.047)
	55–75	1.314 (−2.438–5.065)	3.125 (−0.377–6.627)	−4.739 (−12.173–2.696)	−2.605 (−6.808–1.559)	2.008 (−2.873–6.889)	−4.282 (−13.008–4.444)

Regression coefficients of 36 univariate regressions to determine the relationship of outpatient care access (sufficient vs insufficient) on EQ VAS change (T2-T1) using different strata (age, gender, SES, chronic disease status). Asterisks and bold text denote significant results (p < 0.05). 95% Confidence intervals are shown in parentheses

When comparing effects by chronic disease status, healthcare access significantly influenced HRQoL change (improved EQ-5D-5L index change Δ0.037, CI 0.001, 0.073) for male respondents between 35 and 54 years old with middle SES and no chronic disease (Table 2). In comparison, males of the same age and SES with at least one chronic disease(s) had a negligible change in EQ-5D-5L index (Δ−0.008, CI −0.060, 0.043). Male respondents of the same age with high SES and chronic disease(s) with sufficient outpatient care access as predictor resulted in a deteriorated HRQoL (mean EQ-5D-5L index change Δ−0.062**,** CI −0.166, −0.007). Male respondents between 55 and 75 years old with middle SES and no chronic disease had a statistically significant improved HRQoL (EQ VAS change Δ3.7, CI 0.474, 6.901) with sufficient outpatient care access, whereas male respondents of the same age and SES with chronic disease(s) had a non-significant change in HRQoL (EQ VAS change, Δ1.266, CI −6.253, 3.721), shown in Table 3. For male respondents between 55 and 75 years with low SES and no chronic disease, having sufficient outpatient care access corresponded significantly to a deterioration in EQ VAS (Δ−7.0, CI −12.427, −1.590). For male respondents of the same age and SES but with chronic disease(s), EQ VAS did not change significantly (Δ0.282, CI −9.235, 9.800).

An overall effect of gender on change in HRQoL could not be determined (Tables 2 and 3). There were no statistically significant HRQoL changes for any strata containing female respondents.

When comparing effects by SES for male respondents between 55 and 75 years old, middle SES, and no chronic disease, EQ VAS improved (3.7, CI 0.474, 6.901). For male respondents of the same age with low SES and no chronic disease EQ VAS deteriorated: (−7.0, CI −12.427, −1.590). The difference in EQ VAS between respondents with middle SES and low SES was 10.7, with changes in opposite directions. This statistically significant result was only present in EQ VAS change regressions (Table 3), not in EQ-5D-5L index change (Table 2).

### Multiple linear regression models with outcome HRQoL change

The full factorial general linear models showed no significant interaction effects. Thus, we moved forward with main effect models (Table 4). For EQ-5D-5L index change, significant predictors were chronic disease (Δ0.037, CI 0.004, 0.018), and healthcare access (Δ−0.032, CI −0.02, 0.003) (Table 4). There were no significant predictors in the full model for EQ VAS change, apart from gender (Table 5), which lost significance after removing non-significant variables from the full model (see Tables S15 and S16 for full main effect models).

**Table 4:** Final linear regression model for change in EQ-5D-5L index (T2-T1)

Change in EQ-5D-5L index (T2-T1)						
					95% Confidence Interval	
		Beta	Robust Std. Error	Sig	Lower Bound	Upper Bound
Constant			0.003	0.287	−0.004	0.013
Healthcare access						
	Insufficient (ref)					
	Sufficient	−0.032	0.005	0.009	−0.02	−0.003
Chronic disease status						
	None (ref)					
	One or more chronic diseases	0.037	0.004	0.003	0.004	0.018

F = 8.927, p < 0.001, R^2^ = 0.002

**Table 5:** Final linear regression model for change in EQ VAS (T2-T1)

	Change in EQ VAS (T2-T1)					
					95% Confidence Interval	
		Beta	Robust Std. Error	Sig	Lower Bound	Upper Bound
Constant			0.257	< .001	−1.559	−0.564
Gender						
	Male (ref)					
	Female	−0.023	0.349	0.058	−1.353	0.022

F = 3.602, p = 0.058, R^2^ = 0.001

## Discussion

### Summary

Approximately one in five respondents reported insufficient healthcare access across all countries combined. Respondents with chronic disease, those aged 18–34 and 35–54 years old and respondents from Greece and the UK reported insufficient healthcare access more often compared to their counterparts. Previous studies have shown declines in healthcare access during the COVID-19 pandemic, varying across countries [30]. Declines in healthcare access during the first wave of the COVID-19 pandemic have also been reported for women and people with chronic conditions [31]. One study reported 18.6% of a sample of patients with multiple sclerosis had stopped getting treatment during the COVID-19 pandemic, and 13.4% were unsatisfied with their healthcare [32].

An intriguing finding is the dual association of insufficient healthcare access: such respondents more often reported either better or worse HRQoL compared to respondents with sufficient access, who had a higher percentage of unchanged HRQoL. The percentage of improved HRQoL was slightly higher than of deteriorated HRQoL for the EQ-5D-5L index and slightly higher for deteriorated HRQoL for EQ VAS, regardless of healthcare access category. Previous longitudinal studies on change in HRQoL during the COVID-19 pandemic have yielded mixed results: HRQoL was reported to be negligibly affected during the pandemic in several populations of chronically ill patients and a general population sample of Norwegian parents [32–37], but was found to be significantly deteriorated in certain groups of chronically ill patients as well as in the Japanese and Estonian general populations [38–40]. A study among Chinese adults found mixed change in HRQoL [41]. It should be noted that there was a large variety of follow-up intervals in the aforementioned studies.

We found no evidence of significant HRQoL change that was associated with healthcare access at T1 when stratified by age, gender, SES, and chronic disease status during a 1-year period of the COVID-19 pandemic in the general population of five countries. Stratification resulted in a non-significant effect of healthcare access on HRQoL in almost all strata apart from two, and were likely chance findings. Thus, in this study, background variables (age, gender, SES, and chronic disease status) did not exhibit effect modification in the relationship between healthcare access at T1 and HRQoL change. The context-specific nature of healthcare access in different countries may have contributed to the heterogeneity of our findings, as well as individuals’ perception of unmet healthcare needs [42, 43]. Government stringency of pandemic measures and restrictions varied by country as well as by US state throughout the COVID-19 pandemic, with especially Italy implementing restrictions earlier compared to other countries in Europe and the US [44] [45]. Healthcare system resilience also varied across countries, with outpatient facilities being more affected in some countries compared to others [30, 46]. For example, the median decline in outpatient care services utilization in Italy was reportedly higher than in the US [30].

Another explanation for the lack of strong association of healthcare access with change in HRQoL is the participant selection in this study. This study consisted of the general population, rather than a hospitalized or primary care sample, which relied on self-report of chronic conditions, and potentially led to reporting of fewer chronic conditions. Additionally, there was an underrepresentation of young adults (18–24 year olds) whose HRQoL and mental wellbeing has been reported to be affected substantially by COVID-19 pandemic restrictions [47, 48]. There may also be additional factors that we did not measure that can be attributed to HRQoL change, such as health literacy, marriage status, and diet [39, 40, 49, 50]. Furthermore, mixed results have been reported in the literature regarding change in HRQoL of elderly people. Some studies suggest HRQoL deteriorated for the elderly whereas others suggest it remained the same [51–53]. Likewise, telehealth was reported to offset effects of missed outpatient care visits, whereas another study found that the effect varied greatly across populations with low access to telehealth in disadvantaged communities [54, 55]. On the other hand, the effect of deferred care may not yet show in HRQoL trends but will in the coming years (e.g. with missed cancer screening opportunities due to the COVID-19 pandemic) [56, 57]. Additionally, the timeframe of data collection may explain a discrepancy between our results and published studies. Numerous studies compare HRQoL before the pandemic to HRQoL during early stages of the pandemic (2020), or only examine HRQoL at one point in time [20, 41, 53, 58, 59], whereas our study investigated change between early 2020 and 2021.

Lastly, depression and anxiety increased during the COVID-19 pandemic, especially for younger persons [58, 60], which may indicate that mental health and distress due to the COVID-19 pandemic had a greater association with HRQoL change over a 1-year period than did healthcare access.

### Strengths and limitations

Strengths of this study include its longitudinal and multi-country design. This aspect of the study allowed for a large cohort of respondents with greater heterogeneity of COVID-19 pandemic effects. The longitudinal design allowed for the possibility of investigating the relationship between healthcare access and change in HRQoL over time. Furthermore, the first wave of data collection (T1) occurred in the earliest stage of the COVID-19 pandemic, when healthcare access was first disrupted globally [61, 62]. This allowed us to study the effect of the COVID-19 pandemic on healthcare access in the general population. Furthermore, we were able to separate effects of various variables (i.e., age, gender, SES, and chronic disease status) by stratification.

This study has several limitations. Firstly, although controlling for various variables is a strength, it is also a weakness as it resulted in strata that contained small frequencies of respondents. Due to this corresponding reduced power we could not further stratify respondents by COVID-19 status, to differentiate between effect of chronic disease and COVID-19 infection. Exact numbers of respondents per stratum can be found in Table S7. The multivariate regression model we employed to assess the association between healthcare access at T1 and HRQoL change does not consider heterogeneity within subjects, which may have introduced bias. Secondly, due to the longitudinal nature of the study, there is a possibility of attrition bias. Only 41% of the respondents who completed the survey at T1 also completed the follow-up survey at T2. As a consequence, the results cannot be generalized to populations not consisting primarily of older (55–75 years old) people with a high education. The forced choice design of the questionnaire may have contributed to nonresponse bias, and could have introduced social desirability bias and response set bias. Additionally, there was no extensive information on other aspects of healthcare access, such as access to medication. In the same vein, government stringency of pandemic measures varied per country, as previously mentioned [44, 45]. These factors may have led to an inaccurate portrayal of healthcare access in the early stages of the COVID-19 pandemic. Furthermore, SES was measured only through education attainment due to missing data related to income from 12 participants, and marital status was not measured, both of which may have contributed to loss of information regarding SES. Moreover, we were not able to conduct mediation analysis due to possible residual confounding. We recommend future studies to look into the mediation effect of healthcare access. It should be noted that New York State and New York City were oversampled, and likely had greater access to healthcare as compared to many other regions in the US. Lastly, we dichotomized our chronic disease variable, which may have resulted in a loss of information.

## Conclusion

Approximately one in five respondents reported insufficient access to outpatient care. Respondents with insufficient healthcare access had both more improved HRQoL and deteriorated HRQoL than respondents with sufficient healthcare access, who had a higher percentage of unchanged HRQoL. In this study, age, gender, SES, and chronic disease status did not moderate the relationship between healthcare access at T1 and HRQoL change over a 1-year period during the COVID-19 pandemic when stratified.

## Supplementary Information

Below is the link to the electronic supplementary material.

Supplementary file1 (DOCX 2099 KB)

## Data Availability

Data is available upon reasonable request.
