# Supplementary material for: The relationship between healthcare access and change in health-related quality-of-life among the general population of five countries during the COVID-19 pandemic
Source: Qual Life Res. 2024 Jun 11;33(9):2541–52. doi: 10.1007/s11136-024-03704-1 (PMC11390791; doi:10.1007/s11136-024-03704-1)
Supplement: Supplementary file 1 — Supplementary file1 (DOCX 2099 KB) [file 11136_2024_3704_MOESM1_ESM.docx]

**Supplementary Materials**

Table S2.a. Dropout rate at T1 per country

| **Country** | **Number of respondents started the survey, but never finished** | **Dropout rate** |
| --- | --- | --- |
| Greece | 89 | 8.00% |
| Italy | 124 | 4.70% |
| Netherlands | 446 | 12.60% |
| UK | 312 | 10.40% |
| USA | 723 | 10.0% |

Table S2b. Dropout rate at T2 per country

| **Country** | **Number of respondents started the survey, but never finished** | **Dropout rate** |
| --- | --- | --- |
| Greece | 19 | 3.60% |
| Italy | 136 | 7.10% |
| Netherlands | 165 | 12.40% |
| UK | 136 | 8.4% |
| USA | 230 | 10.6% |

Table S1. Baseline characteristics included in Table 1 and accompanying grouping method.

| **Variable** | **Description** |
| --- | --- |
| Age category | Age was an open question. For analysis purposes, the following age categories were created:  18-34 years old  35-54 years old  55-75 years old |
| Chronic disease status | 11 different chronic conditions were included in the questionnaire, as well as an open response text box for “other”. The conditions included the following: asthma or chronic bronchitis, heart disease/failure, diabetes, cancer, lung emphysema, stroke, rheumatoid arthritis, severe back complaints, arthrosis, memory problems, and depression or anxiety disorder. |
| Living situation | Living situation had the following response categories: “living alone”, “living with a partner without children”, “living alone with one or more children”, “living with my parents, without children”, “living with my parents, with one or more children”, “living with my parents and partner, with one or more children”, and “other”. Responses were categorized into the following options: living with others, living alone, and other. |
| Sex | Sex at T1 was a dichotomous variable, categorized into “female” or “male”. |
| Education level | Education level response levels varied by country. The responses were categorized into three levels according to ISCED classification: low, middle, and high. |
| Occupation | Occupation had the following response categories: “in work: employee”, “In work: self-employed”, “out of work for more than 1 year”, “out of work for less than 1 year”, “looking after others (e.g., caretaker or parent), “student”, “retired”, and “unable to work”. The responses were categorized into the following categories: employed, unemployed, student, retired, and unable to work. |
| Healthcare access | Healthcare access was assessed through outpatient care access. Access was related to ease of getting an appointment, waiting times, and general experience was regard to access. Outpatient care access was an ordinal variable with two versions of 5 levels: always good, usually good, sometimes good, usually not good, and never good, and for the other version: very good, good, fair, bad, and very bad. For analysis purposes, outpatient care access was dichotomized into “sufficient” (always good, usually good and very good, good) versus “insufficient” (sometimes good, usually not good, never good and fair, bad, very bad). |
| COVID-19 infection | COVID-19 infection was assessed at T1 using the following response categories:  “I do not have a coronavirus infection, I have not had a fever and I have not been in contact with anyone ill”,  “I may have been infected with coronavirus because I have a slight fever or cough. I have not been tested”,  “I may have been infected with corona because I have been in contact with someone who later turned out to be ill”, “I may have been infected due to symptoms of coronavirus or I have had a positive test”, “I have been infected with coronavirus and am (almost) completely recovered”, “I have been infected with coronavirus and am still experiencing moderate to severe health problems” , and was categorized into “infected” versus “not infected”. The latter three categories were grouped together and deemed “infected”. |

Table S3. Baseline characteristics for respondents from each country at T1, divided by outpatient care access (sufficient versus insufficient).

|  | | Greece | | Italy | | | | Netherlands | | | | UK | | | | US | | | | Total | | | |  |
| --- | --- | --- | --- | --- | --- | --- | --- | --- | --- | --- | --- | --- | --- | --- | --- | --- | --- | --- | --- | --- | --- | --- | --- | --- |
| Response rate (T2/T1) | | 50% | | 56% | | | | 35% | | | | 45% | | | | 32% | | | | 41% | | | |  |
| Number of respondents | | 511 | | 1784 | | | | 1143 | | | | 1448 | | | | 1879 | | | | 6765 | | | |  |
| Baseline characteristics (T1) | |  |  | |  |  |  | |  | |  | |  | |  | |  | |  | |  | |  | |
| Outpatient care access | | insufficient | sufficient | insufficient | | sufficient | | insufficient | | sufficient | | insufficient | | sufficient | | insufficient | | sufficient | | insufficient | | sufficient | |  |
| Number of respondents per outpatient care access category | | 149 (29.2%) | 362. (70.8) | 431 (24.2%) | | 1353 (75.8%) | | 155 (13.6%) | | 988 (86.4%) | | 384 (26.5%) | | 1064 (73.5%) | | 219 (11.7%) | | 1660 (88.3%) | | 1338 (19.8%) | | 5427 (80.2%) | |  |
| Age | |  |  | |  |  |  | |  | |  | |  | |  | |  | |  | |  | |  | |
| Median (IQR) | | 43 (20) | 43 (18) | 44 (19) | | 46(22) | | 47(24) | | 58(20) | | 51(23) | | 54(23) | | 49(21) | | 57(20) | | 47(21) | | 53(23) | |  |
|  |  |  |  |  |  |  |  |  |  |  |  |  |  |  |  |  |  |  |  |  |  |  |  |  |
| Mean (SD) | | 43.62 (12.39) | 43.31 (12.59) | 44.92 (12.54) | | 47.35 (-14.02) | | 47.86 (-14.31) | | 55.04 (-13.57) | | 49.41 (-14.33) | | 52.26 (-14.11) | | 47.86 (-12.96) | | 55.15 (-12.79) | | 46.76 (13.52) | | 51.85 (14.03) | |  |
|  |  |  |  |  |  |  |  |  |  |  |  |  |  |  |  |  |  |  |  |  |  |  |  |  |
|  |  |  |  |  |  |  |  |  |  |  |  |  |  |  |  |  |  |  |  |  |  |  |  |  |
| Age group | 18-34 | 46 (30.9%) | 88 (24.3%) | 98 (22.7%) | | 272 (20.1%) | | 33 (21.3%) | | 91 (9.2%) | | 74 (19.3%) | | 144 (13.5%) | | 40 (18.3%) | | 127 (7.7%) | | 291 (21.7%) | | 722 (13.3%) | |  |
|  | 35-54 | 75 (50.3%) | 188 (51.9%) | 236 (54.8%) | | 641 (47.4%) | | 65 (41.9%) | | 334 (33.8%) | | 145 (37.8%) | | 409 (38.4%) | | 105 (47.9%) | | 607 (36.6%) | | 626 (46.8%) | | 2179 (40.2%) | |  |
|  | 55-75 | 28 (18.8%) | 86 (23.8%) | 97 (22.5%) | | 440 (32.5%) | | 57 (36.8%) | | 563 (57.0%) | | 165 (43.0%) | | 511 (48.0%) | | 74 (33.8%) | | 926 (55.8%) | | 421 (31.5%) | | 2526 (46.5%) | |  |
| Sex | Male | 83 (55.7%) | 180 (49.7%) | 206 (47.8%) | | 653 (48.3%) | | 58 (37.4%) | | 468 (47.4%) | | 191 (49.7%) | | 518 (48.7%) | | 88 (40.2%) | | 781 (47.0%) | | 626 (46.8%) | | 2600 (47.9%) | |  |
|  | Female | 66 (44.3%) | 182 (50.3%) | 225 (52.2%) | | 700 (51.7%) | | 97 (62.6%) | | 520 (52.6%) | | 193 (50.3%) | | 546 (51.3%) | | 131 (59.8%) | | 879 (53.0%) | | 712 (53.2%) | | 2827 (52.1%) | |  |
| Occupation status | Employed | 82 (55.0%) | 207 (57.2%) | 242 (56.1%) | | 766 (56.6%) | | 85 (54.8%) | | 467 (47.3%) | | 210 (54.7%) | | 587 (55.2%) | | 122 (55.7%) | | 854 (51.4%) | | 741 (55.4%) | | 2881 (53.1%) | |  |
|  | Student | 10 (6.7%) | 21 (5.8%) | 19 (4.4%) | | 58 (4.3%) | | 6 (3.9%) | | 28 (2.8%) | | 4 (1.0%) | | 13 (1.2%) | | 3 (1.4%) | | 12 (0.7%) | | 42 (3.1%) | | 132 (2.4%) | |  |
|  | Unemployed | 46 (30.9%) | 88 (24.3%) | 116 (26.9%) | | 299 (22.1%) | | 22 (14.2%) | | 102 (10.3%) | | 54 (14.1%) | | 106 (10.0%) | | 45 (20.5%) | | 189 (11.4%) | | 283 (21.2%) | | 784 (14.4%) | |  |
|  | Retired | 11 (7.4%) | 41 (11.3%) | 49 (11.4%) | | 220 (6.3%) | | 22 (14.2%) | | 283 (28.6%) | | 73 (19.0%) | | 292 (27.4%) | | 26 (11.9%) | | 533 (32.1%) | | 181 (13.5%) | | 1369 (25.2%) | |  |
|  | Unable to work | 0 (0.0%) | 5 (1.4%) | 5 (1.2%) | | 10 (0.7%) | | 20 (12.9%) | | 108 (10.9%) | | 43 (11.2%) | | 66 (6.2%) | | 23 (10.5%) | | 72 (4.3%) | | 91 (6.8%) | | 261 (4.8%) | |  |
| Education level | High | 96 (64.4%) | 247 (68.2%) | 189 (43.9%) | | 537 (39.7%) | | 76 (49.0%) | | 388 (39.3%) | | 209 (54.4%) | | 598 (56.2%) | | 159 (72.6%) | | 1234 (74.3%) | | 729 (54.5%) | | 3004 (55.4%) | |  |
|  | Middle | 47 (31.5%) | 106 (29.3%) | 171 (39.7%) | | 615 (45.5%) | | 39 (25.2%) | | 312 (31.6%) | | 164 (42.7%) | | 444 (41.7%) | | 50 (22.8%) | | 384 (23.1%) | | 471 (35.2%) | | 1861 (34.3%) | |  |
|  | Low | 6 (4.0%) | 9 (2.5%) | 71 (16.5%) | | 201 (14.9%) | | 40 (25.8%) | | 288 (29.1%) | | 11 (2.9%) | | 22 (2.1%) | | 10 (4.6%) | | 42 (2.5%) | | 138 (10.3%) | | 562 (10.4%) | |  |
| COVID-19 status | Not infected | 146 (98.0%) | 361 (99.7%) | 424 (98.4%) | | 1332 (98.4%) | | 150 (96.8%) | | 965 (97.7%) | | 373 (97.1%) | | 1048 (98.5%) | | 199 (90.9%) | | 1624 (97.8%) | | 1292 (96.6%) | | 5330 (98.2%) | |  |
|  | Infected | 3 (2.0%) | 1 (0.3%) | 7 (1.6%) | | 21 (1.6%) | | 5 (3.2%) | | 23 (2.3%) | | 11 (2.9%) | | 16 (1.5%) | | 20 (9.1%) | | 36 (2.2%) | | 46 (3.4%) | | 97 (1.8%) | |  |
| Chronic disease status | None | 81 (54.4%) | 223 (61.6%) | 237 (55.0%) | | 891 (65.9%) | | 70 (45.2%) | | 475 (48.1%) | | 206 (53.6%) | | 624 (58.6%) | | 118 (53.9%) | | 963 (58.0%) | | 712 (53.2%) | | 3176 (58.5%) | |  |
|  | One or more | 68 (45.6%) | 139 (38.4%) | 194 (45.0%) | | 462 (34.1%) | | 85 (54.8%) | | 513 (51.9%) | | 178 (46.4%) | | 440 (41.4%) | | 101 (46.1%) | | 697 (42.0%) | | 626 (46.8%) | | 2251 (41.5%) | |  |
| Living situation | Living with others | 120 (80.5%) | 310 (85.6%) | 383 (88.9%) | | 1206 (89.1%) | | 108 (69.7%) | | 689 (69.7%) | | 286 (74.5%) | | 829 (77.9%) | | 142 (64.8%) | | 1271 (76.6%) | | 1039 (77.7%) | | 4305 (79.3%) | |  |
|  | Living alone | 25 (16.8%) | 49 (13.5%) | 42 (9.7%) | | 127 (9.4%) | | 44 (28.4%) | | 289 (29.3%) | | 90 (23.4%) | | 215 (20.2%) | | 62 (28.3%) | | 345 (20.8%) | | 263 (19.7%) | | 1025 (18.9%) | |  |
|  | Other | 4 (2.7%) | 3 (0.8%) | 6 (1.4%) | | 20 (1.5%) | | 3 (1.9%) | | 10 (1.0%) | | 8 (2.1%) | | 20 (1.9%) | | 15 (6.8%) | | 44 (2.7%) | | 36 (2.7%) | | 97 (1.8%) | |  |

Table S4. Outpatient care access per age category based on country, percentages by row shown. Age category at T1 is shown.

|  |  | Outpatient care access | | | |
| --- | --- | --- | --- | --- | --- |
|  |  | Insufficient | | Sufficient | |
| Country | Age category at T1 | N | Row N % | N | Row N % |
| Greece | 18-34 | 46 | 34.30% | 88 | 65.70% |
|  | 35-54 | 75 | 28.50% | 188 | 71.50% |
|  | 55-75 | 28 | 24.60% | 86 | 75.40% |
| Italy | 18-34 | 98 | 26.50% | 272 | 73.50% |
|  | 35-54 | 236 | 26.90% | 641 | 73.10% |
|  | 55-75 | 97 | 18.10% | 440 | 81.90% |
| Netherlands | 18-34 | 33 | 26.60% | 91 | 73.40% |
|  | 35-54 | 65 | 16.30% | 334 | 83.70% |
|  | 55-75 | 57 | 9.20% | 563 | 90.80% |
| UK | 18-34 | 74 | 33.90% | 144 | 66.10% |
|  | 35-54 | 145 | 26.20% | 409 | 73.80% |
|  | 55-75 | 165 | 24.40% | 511 | 75.60% |
| US | 18-34 | 40 | 24.00% | 127 | 76.00% |
|  | 35-54 | 105 | 14.70% | 607 | 85.30% |
|  | 55-75 | 74 | 7.40% | 926 | 92.60% |
| Total | 18-34 | 291 | 28.70% | 722 | 71.30% |
|  | 35-54 | 626 | 22.30% | 2179 | 77.70% |
|  | 55-75 | 421 | 14.30% | 2526 | 85.70% |

Table S5. Baseline characteristics for all respondents at T1, divided by outpatient care access (sufficient versus insufficient) with percentages shown per column.

|  |  |  | Outpatient care access | |
| --- | --- | --- | --- | --- |
| Response rate (T2/T1) | |  | 6765 (41%) | |
|  |  | Total | Sufficient | Insufficient |
|  |  |  |  |  |
| Number of respondents |  | 6765 (100%) | 5427 (80.2%) | 1338 (19.8%) |
| Age category |  |  |  |  |
|  | Median (IQR) | 51.00 (23.00) | 53.0 (23.0) | 46.0 (21.0) |
|  | Mean (SD) | 50.84 (14.08) | 51.9 (14.0) | 46.8 (13.5) |
|  | 18-34 | 1013 (15.0%) | 722 (13.3%) | 291 (21.7%) |
|  | 35-54 | 2805 (41.5%) | 2179 (40.2%) | 626 (46.8%) |
|  | 55-75 | 2947 (43.6%) | 2526 (46.5%) | 421 (31.5%) |
| Gender | Male | 3226 (47.7%) | 2600 (47.9%) | 626 (46.8%) |
|  | Female | 3539 (52.3%) | 2827 (52.1%) | 712 (53.2%) |
| Living situation | Living with others | 5344 (79.0%) | 4305 (79.3%) | 1039 (77.7%) |
|  | Living alone | 1288 (19.0%) | 1025 (18.9%) | 263 (19.7%) |
|  | Other | 133 (2.0%) | 97 (1.8%) | 36 (2.7%) |
| Education | High | 3733 (55.2%) | 3004 (55.4%) | 729 (54.5%) |
|  | Middle | 2332 (34.5%) | 1861 (34.3%) | 471 (35.2%) |
|  | Low | 700 (10.3%) | 562 (10.4%) | 138 (10.3%) |
| Chronic disease status | No chronic disease | 3888 (57.5%) | 3176 (58.5%) | 712 (53.2%) |
|  | One or more chronic disease(s) | 2877 (42.5%) | 2251 (41.5%) | 626 (46.8%) |
| COVID-19 infection status | Not infected | 6622 (97.9%) | 5330 (98.2%) | 1292 (96.6%) |
|  | Infected | 143 (2.1%) | 97 (1.8%) | 46 (3.4%) |
| Occupation status | Employed | 3622 (53.5%) | 2881 (53.1%) | 741 (55.4%) |
|  | Student | 174 (2.6%) | 132 (2.4%) | 42 (3.1%) |
|  | Unemployed | 1067 (15.8%) | 784 (14.4%) | 283 (21.2%) |
|  | Retired | 1550 (22.9%) | 1369 (25.2%) | 181 (13.5%) |
|  | Unable to work | 352 (5.2%) | 261 (4.8%) | 91 (6.8%) |
| Country | Greece | 511 (7.6%) | 362 (6.7%) | 149 (11.1%) |
|  | Italy | 1784 (26.4%) | 1353 (24.9%) | 431 (32.2%) |
|  | Netherlands | 1143 (16.9%) | 988 (18.2%) | 155 (11.6%) |
|  | UK | 1448 (21.4%) | 1064 (19.6%) | 384 (28.7%) |
|  | US | 1879 (27.8%) | 1660 (30.6%) | 219 (16.4%) |

Table S6. Univariate regression showing Healthcare access on EQ-5D-5L index (T2-T1) and EQ VAS (T2-T1).

|  | Unstandardized Coefficients |  | 95 % Confidence Interval | |
| --- | --- | --- | --- | --- |
|  | B | Std. Error | Lower Bound | Upper Bound |
| EQ-5D-5L index | **-0.012 *** | 0.004 | **-0.021** | **-0.003** |
| EQ VAS | -0.253 | 0.44 | -1.116 | 0.609 |


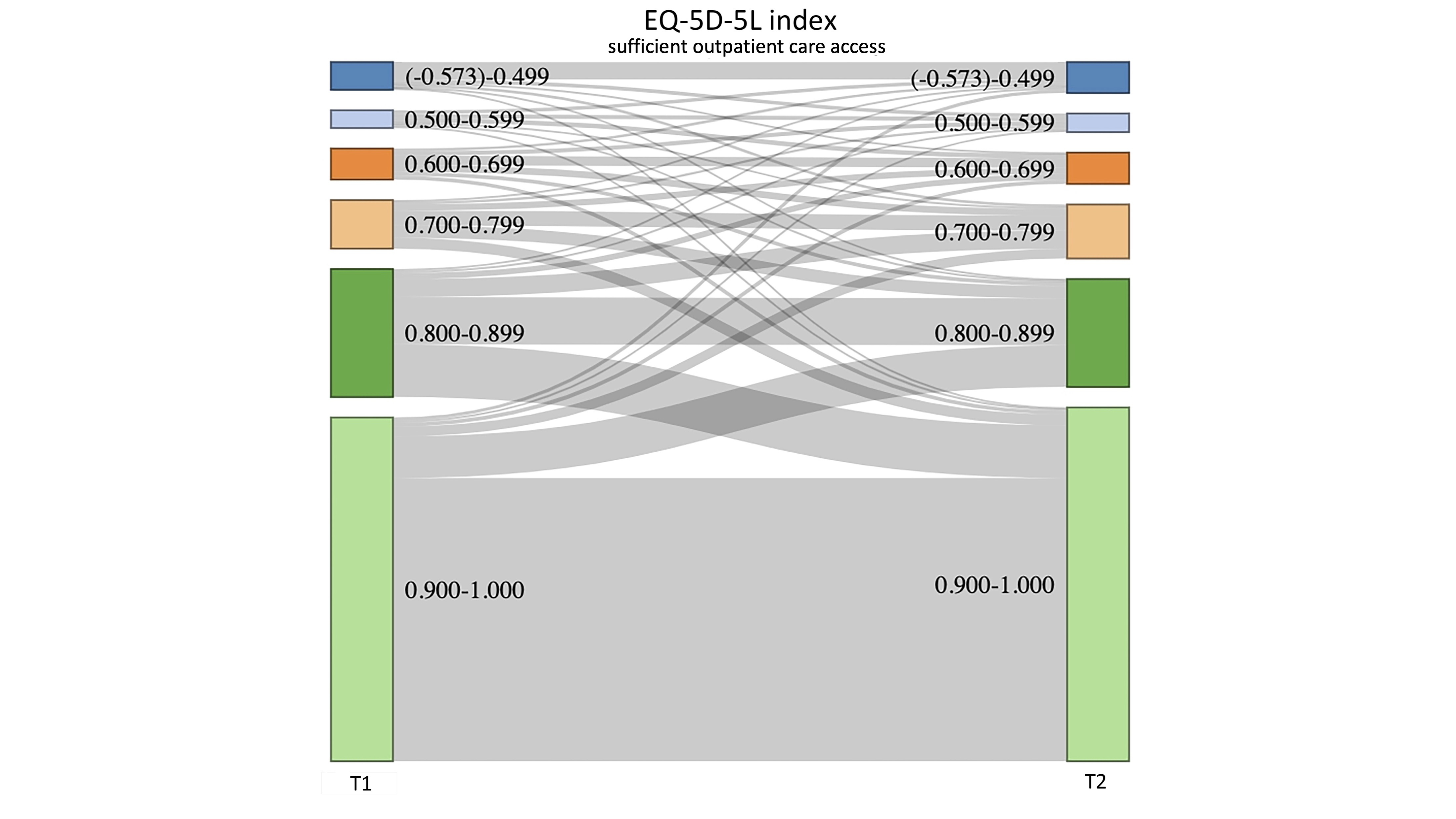

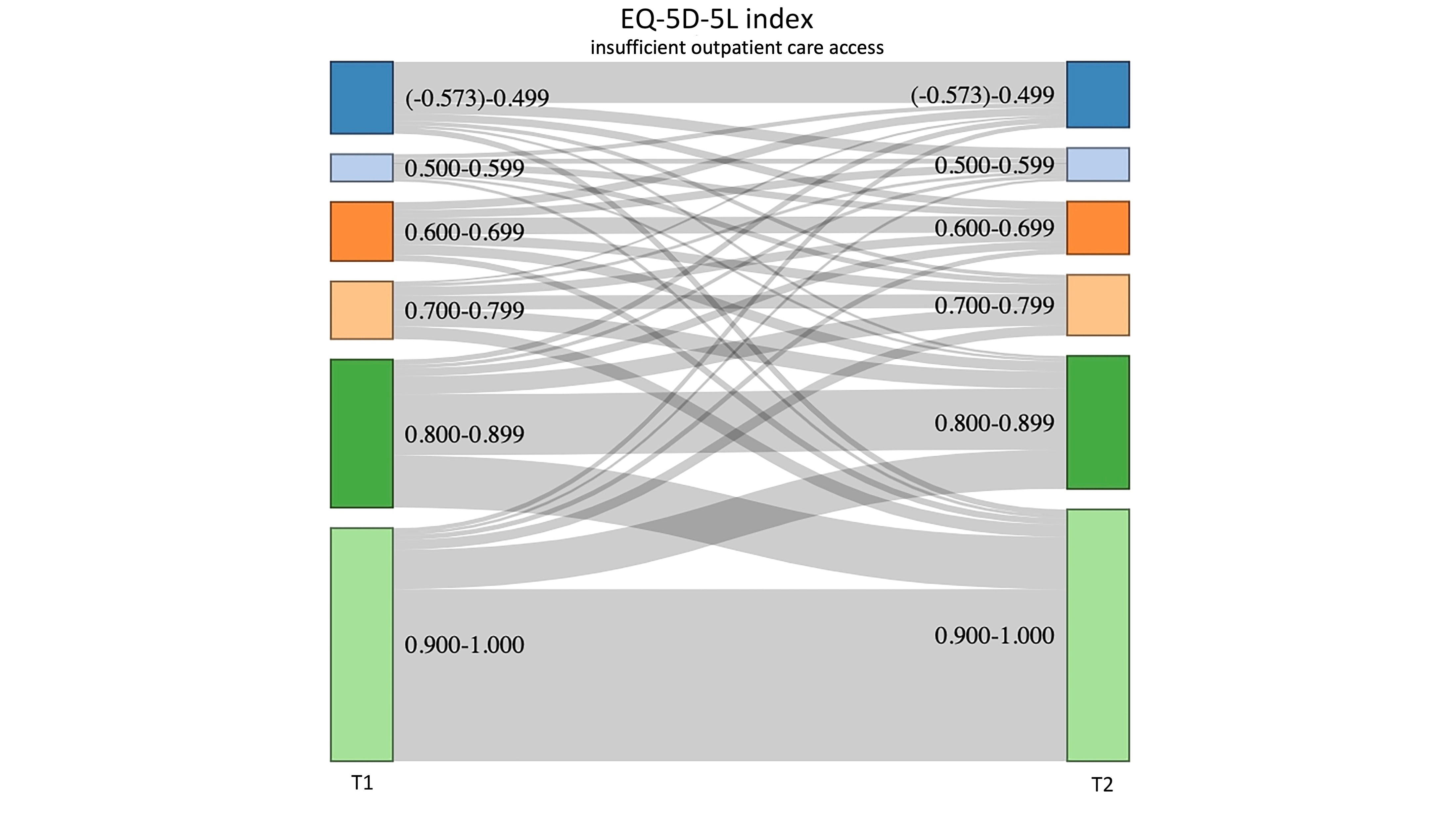


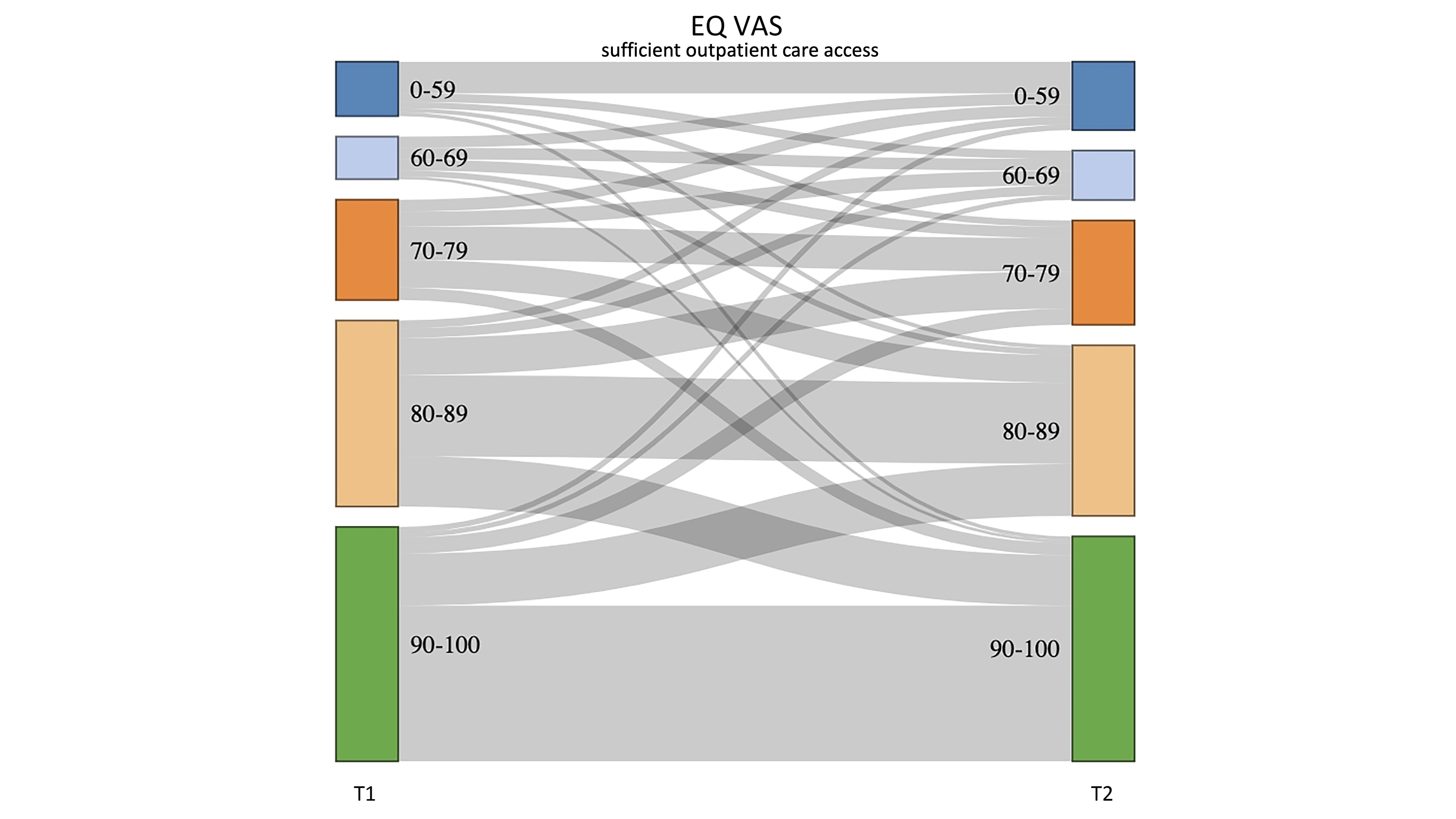

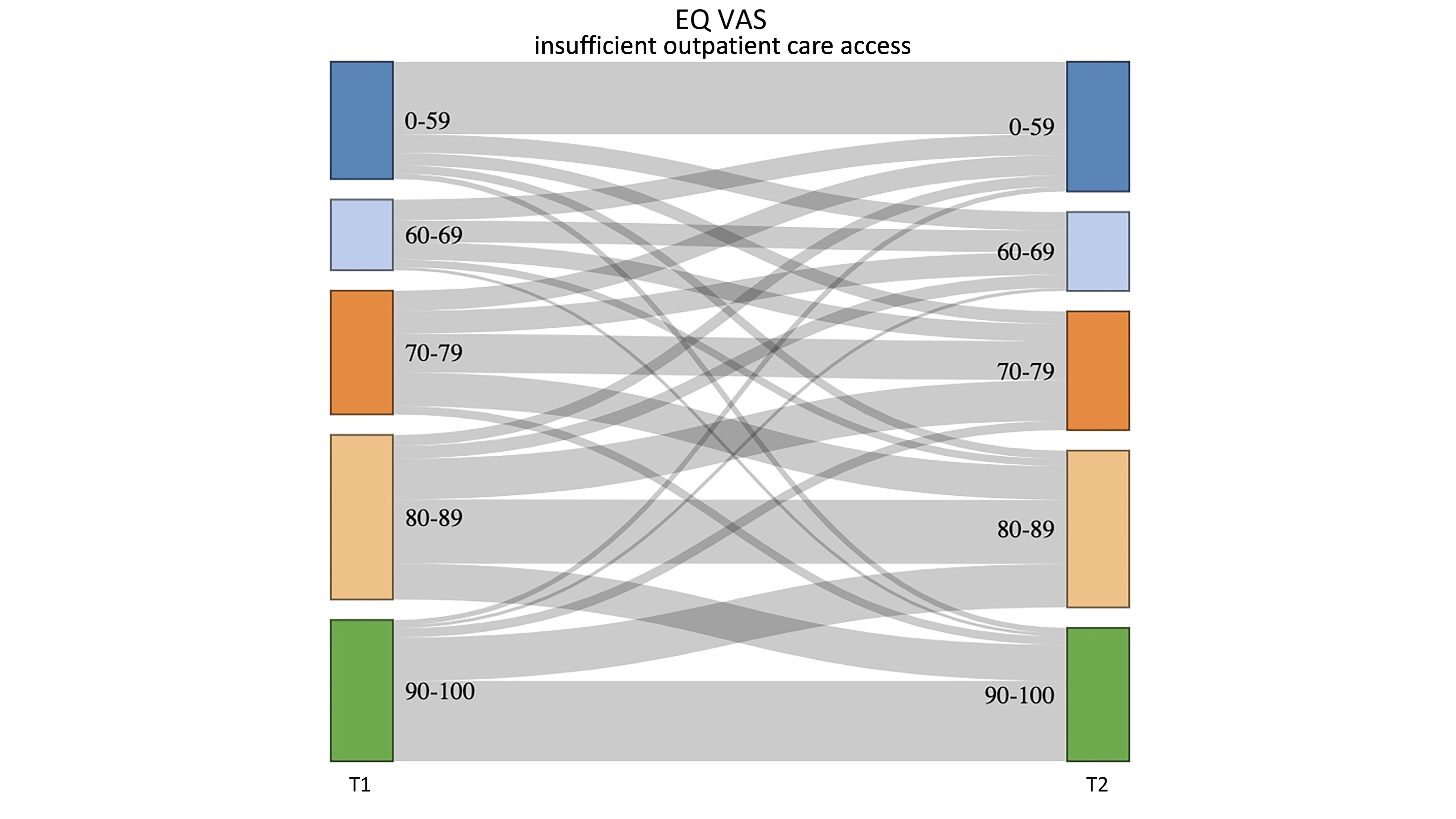


Figure S1a. Changes in EQ-5D-5L index and EQ VAS, stratified by outpatient care access (sufficient versus insufficient). From top left to bottom right: change in EQ-5D-5L index for sufficient access, change in EQ-5D-5L index for insufficient access, change in EQ VAS for sufficient access, change in EQ VAS for insufficient access. EQ-5D-5L index was divided into categories: (-0.573)-0.499, 0.500-0.599, 0.600-0.699, 0.700-0.799, 0.800-0.899, and 0.900-1.000. EQ VAS was divided into categories: 0-59, 60-69, 70-79, 80-89, 90-100.

Figure S1b. Changes in EQ-5D-5L index and EQ VAS between T1 and T2 by sufficient vs. insufficient Healthcare Access for the Total Population. Scores improved if T2 – T1 was a positive number, deteriorated if T2 – T1 was negative, and remained unchanged if T2 – T1 was equal to 0.

*S3. Changes in HRQoL by Outpatient Healthcare Access for the total population*

Figure 1a depicts the flow of change in EQ-5D-5L index and EQ VAS. Cross-sectionally, at T1 and T2 the percentage of respondents with full health according to the EQ-5D-5L index and EQ VAS was higher among those with sufficient outpatient care access compared to those with insufficient outpatient care access. The mean change in HRQoL between the two categories was statistically significant for the EQ-5D-5L index, but not for EQ VAS (EQ-5D-5L index: change (Δ):-0.012, 95% CI: -0.021 - -0.003, EQ VAS: Δ-0.253, 95% CI: -1.116 – 0.609), shown in Table S5.

Overall, a higher proportion of respondents with insufficient outpatient care access experienced change (both positive and negative) in EQ-5D-5L index and EQ VAS scores than respondents with optimal outpatient care access (Figure 1b). Improvement in EQ-5D-5L index scores was more frequent than deterioration (38.0% versus 34.4%) for respondents with insufficient outpatient care access. For EQ VAS, deterioration was more frequent (41.3% versus 35.2%).

Table S7. Frequencies of respondents in each stratum (each stratum consists of sex, age, SES, and chronic disease status) used in regression analysis.

|  |  | Chronic disease status | | | | | | |
| --- | --- | --- | --- | --- | --- | --- | --- | --- |
|  |  | No chronic disease | | | One or more chronic disease(s) | | | |
|  |  | SES | | | SES | | | |
| Gender | Age | High | Middle | Low | | High | Middle | Low |
| Male | 18-34 | 141 | 82 | 15 | | 64 | 40 | 5 |
|  | 35-54 | 519 | 274 | 56 | | 263 | 151 | 39 |
|  | 55-75 | 444 | 264 | 74 | | 432 | 268 | 95 |
| Female | 18-34 | 298 | 116 | 14 | | 149 | 78 | 11 |
|  | 35-54 | 544 | 308 | 86 | | 299 | 200 | 66 |
|  | 55-75 | 303 | 256 | 94 | | 277 | 295 | 145 |

Table S8. Regression coefficients of 12 univariate regressions to determine the relationship of outpatient care access (insufficient vs sufficient) on EQ-5D-5L index change (T2-T1) using different strata (sex, age, chronic disease). Asterisks and bold text denote significant result (p<0.05).

|  |  | Chronic disease status | |
| --- | --- | --- | --- |
| Gender | Age Category | No chronic disease | One or more chronic disease(s) |
| Male | 18-34 | -0.047 (-0.095 – 0.002) | 0.035 (-0.062 – 0.132) |
|  | 35-54 | 0.000 (-0.025 – 0.025) | **-0.043* (-0.080 - -0.006)** |
|  | 55-75 | 0.013 (-0.004 – 0.030) | -0.006 (-0.033 – 0.020) |
| Female | 18-34 | -0.006 (-0.041 – 0.029) | -0.016 (-0.076 – 0.043) |
|  | 35-54 | -0.009 (-0.032 – 0.013) | -0.009 (-0.042 – 0.024) |
|  | 55-75 | -0.007 (-0.028 – 0.014) | -0.020 (-0.050 – 0.010) |

Table S9. Regression coefficients of 12 univariate regressions to determine the relationship of outpatient care access (insufficient vs sufficient) on EQ-5D-5L VAS change (T2-T1) using different strata (sex, age, chronic disease). Asterisks and bold text denote significant result (p<0.05).

|  |  | Chronic disease status | |
| --- | --- | --- | --- |
| Gender | Age category | No chronic disease | One or more chronic disease(s) |
| Male | 18-34 | -1.548 (-5.938 – 2.841) | 5.664 (-2.067 – 13.394) |
|  | 35-54 | 0.207 (-1.980 – 2.394) | **-3.279* (-6.245 – -0.313)** |
|  | 55-75 | 1.236 (-0.938 – 3.411) | -1.594 (-4.369 – 1.181) |
| Female | 18-34 | 0.741 (-2.805 – 4.288) | -0.829 (-5.645 – 3.987) |
|  | 35-54 | 1.215 (-1.266 – 3.696) | 0.326 (-3.092 – 3.743) |
|  | 55-75 | 0.893 (-1.566 – 3.352) | -1.321 (-4.336 – 1.694) |

Table S10. Regression coefficients of 18 univariate regressions to determine the relationship of outpatient care access (insufficient vs sufficient) on EQ-5D-5L index change (T2-T1) using different strata (age, SES, and chronic disease) for respondents whose last outpatient care visit was ≤ 3 months ago. Asterisks and bold text denote significant result (p<0.05).

| EQ-5D-5L index (last visit ≤ 3 months ago) | | | | | | |
| --- | --- | --- | --- | --- | --- | --- |
|  | Chronic disease status | | | | | |
|  | No chronic disease | | | One or more chronic disease(s) | | |
|  |  |  |  |  |  |  |
|  | SES |  |  | SES | |  |
| Age | High | Mid | Low | High | Mid | Low |
| **18-34** | **-0.092* (-0.183 - -0.002)** | -0.108 (-0.290 - 0.071) | **0.445* (0.040 - 0.849)** | 0.081 (-0.013 - 0.176) | 0.037 (-0.105 - 0.179) | 0.056 (-0.361 - 0.473) |
| **35-54** | -0.008 (-0.074) - 0.058) | -0.038 (-0.108 - 0.031) | -0.033 (-0.143 - 0.077) | **-0.071* (-0.135 - - 0.007)** | -0.014 (-0.075 - 0.048) | -0.072 (-0.231 - 0.087) |
| **55-75** | 0.045 (-0.001 - 0.091) | 0.060 (-0.011 - 0.130) | -0.019 (-0.112 - 0.074) | -0.024 (-0.068 - 0.020) | -0.006 (-0.063 - 0.050) | -0.008 (-0.083 - 0.068) |

Table S11. Regression coefficients of 18 univariate regressions to determine the relationship of outpatient care access (insufficient vs sufficient) on EQ VAS change (T2-T1) using different strata (age, SES, and chronic disease) for respondents whose last outpatient care visit was ≤ 3 months ago. Asterisks and bold text denote significant result (p<0.05).

| EQ VAS (last visit ≤ 3 months ago) | | | | | | |
| --- | --- | --- | --- | --- | --- | --- |
|  | Chronic disease status | | | | | |
|  | No chronic disease | | | One or more chronic disease(s) | | |
|  |  |  |  |  |  |  |
|  | SES |  |  | SES | |  |
| Age | High | Mid | Low | High | Mid | Low |
| **18-34** | -3.209 (-11.581 - 5.163) | -4.750 (-15.326 - 5.826) | -30.833 (-61.772 - 0.106) | 0.820 (-7.174 - 8.813) | 4.426 (-7.331 - 16.183) | -3.000 (-20.569 - 14.569) |
| **35-54** | 2.548 (-2.641 - 7.738) | 0.900 (-7.191 - 8.991) | -1.591 (-12.298) | -3.070 (-8.575 - 2.435) | 2.799 (-3.110 - 8.707) | -10.822 (-24.614 - 2.969) |
| **55-75** | **6.508* (0.233 - 12.783)** | 1.348 (-4.644 - 7.340) | -8.600 (-22.824 - 5.624) | **-6.355* (-10.609 - -2.101)** | 1.522 (-4.306 - 7.349) | -5.199 (-14.885 - 4.487 |

Table S12. Regression coefficients of 18 univariate regressions to determine the relationship of outpatient care access (insufficient vs sufficient) on EQ-5D-5L index change (T2-T1) using different strata (age, SES, and chronic disease) for respondents whose last outpatient care visit was > 3 months ago. Asterisks and bold text denote significant result (p<0.05). 95% Confidence intervals shown in parentheses.

| EQ-5D-5L index (last visit > 3 months ago) | | | | | | |
| --- | --- | --- | --- | --- | --- | --- |
|  | Chronic disease status | | | | | |
|  | No chronic disease | | | One or more chronic disease(s) | | |
|  | SES |  |  | SES | |  |
| Age | High | Mid | Low | High | Mid | Low |
| 18-34 | 0.003 (-0.031 – 0.037) | -0.012 (-0.066 – 0.042) | 0.000 (-0.107 – 0.106) | -0.048 (-0.144 – 0.047) | -0.053 (-0.147 – 0.041) | 0.079 (-0.326 – 0.485) |
| 35-54 | -0.017 (-0.039 – 0.004) | **0.032* (0.002 – 0.062)** | -0.001 (-0.091 – 0.089) | -0.028 (-0.067 – 0.010) | 0.008 (-0.039 – 0.056) | 0.038 (-0.054 – 0.129) |
| 55-75 | -0.014 (-0.035 – 0.008) | 0.011 (-0.010 – 0.032) | -0.017 (-0.057 – 0.023) | 0.001 (-0.033 – 0.034) | 0.024 (-0.071 – 0.023) | -0.062 (-0.137 – 0.013) |

Table S13. Regression coefficients of 18 univariate regressions to determine the relationship of outpatient care access (insufficient vs sufficient) on EQ VAS change (T2-T1) using different strata (age, SES, and chronic disease) for respondents whose last outpatient care visit was > 3 months ago. Asterisks and bold text denote significant result (p<0.05). 95% Confidence intervals shown in parentheses.

| EQ VAS (last visit > 3 months ago) | | | | | | | | |
| --- | --- | --- | --- | --- | --- | --- | --- | --- |
|  | Chronic disease status | | | | | | | |
|  | No chronic disease | | | | One or more chronic disease(s) | | | |
|  |  |  |  |  |  |  |  |  |
|  | SES | |  |  | SES | | |  |
| Age | High | | Mid | Low | High | | Mid | Low |
| **18-34** | 1.893 (-2.586 – 4.862) | 1.336 (-4.408 – 7.079) | | 0.500 (-15.568 – 16.568) | 0.300 (-6.476 – 7.076) | 0.764 (-8.435 – 9.964) | | 11.250 (-58.027 – 80.527) |
| **35-54** | -0.750 (-3.027 – 1.528) | | 3.082 (-0.132 – 6.296) | 1.450 (-5.481 – 8.381) | -1.620 (-5.268 – 2.029) | | -3.317 (-8.213 – 1.578) | -1.288 (-10.988 – 8.411) |
| **55-75** | -0.404 (-2.984 – 2.177) | | **3.709* (1.111 – 6.307)** | -4.524 (-9.670 – 0.621) | -0.532 (-4.017 – 2.954) | | -0.340 (-4.685 – 4.005) | -0.229 (-8.952 – 8.494) |

Figure S2. Change in mean EQ-5D-5L index by outpatient care access, last outpatient care visit was ≤ 3 months ago (*change in mean EQ-5D-5L index was significant between sufficient and insufficient outpatient care access categories, p=0.018, one-way ANOVA, p=0.259 for EQ VAS).

Table S14. One-way ANOVA to test for differences in mean change of EQ-5D-5L index and EQ VAS (T2-T1) with respect to outpatient care access when last visit was ≤ 3 months ago.

| ANOVA | | | | | | |
| --- | --- | --- | --- | --- | --- | --- |
|  | | Sum of Squares | df | Mean Square | F | Sig. |
| EQ-5D-5L index T2-T1 | Between Groups | .155 | 1 | .155 | 5.572 | .018 |
|  | Within Groups | 61.791 | 2220 | .028 |  |  |
|  | Total | 61.947 | 2221 |  |  |  |
| EQ VAS T2-T1 | Between Groups | 304.875 | 1 | 304.875 | 1.273 | .259 |
|  | Within Groups | 531826.168 | 2220 | 239.561 |  |  |
|  | Total | 532131.043 | 2221 |  |  |  |

Figure S3. Change in mean EQ VAS by outpatient care access, last outpatient care visit was > 3 months ago (no significant differences between change in mean HRQoL of outpatient care access categories, p=0.084 for EQ-5D-5L index and p=0.971 for EQ VAS).

Table S15. One-way ANOVA to test for differences in mean change of EQ-5D-5L index and EQ VAS (T2-T1) with respect to outpatient care access when last visit was > 3 months ago.

| ANOVA | | | | | | |
| --- | --- | --- | --- | --- | --- | --- |
|  | | Sum of Squares | df | Mean Square | F | Sig. |
| EQ-5D-5L index T2-T1 | Between Groups | .155 | 1 | .155 | 5.572 | .018 |
|  | Within Groups | 61.791 | 2220 | .028 |  |  |
|  | Total | 61.947 | 2221 |  |  |  |
| EQ VAS T2-T1 | Between Groups | 304.875 | 1 | 304.875 | 1.273 | .259 |
|  | Within Groups | 531826.168 | 2220 | 239.561 |  |  |
|  | Total | 532131.043 | 2221 |  |  |  |

Table S16. Full model including non-significant predictors for EQ-5D-5L index change (T2-T1).

|  |  |  |  | 95% Confidence Interval | |
| --- | --- | --- | --- | --- | --- |
|  | Beta | Std. Error | Sig. | Lower Bound | Upper Bound |
| (Constant) |  | 0.006 | 0.829 | -0.012 | 0.01 |
| **Age category** |  |  |  |  |  |
| 18-34 | 0.013 | 0.006 | 0.337 | -0.006 | 0.016 |
| 35-54 | 0.019 | 0.004 | 0.146 | -0.002 | 0.014 |
| 55-75 (ref) |  |  |  |  |  |
| **SES** |  |  |  |  |  |
| High (ref) |  |  |  |  |  |
| Low | 0.006 | 0.006 | 0.638 | -0.009 | 0.015 |
| Middle | 0.009 | 0.004 | 0.48 | -0.005 | 0.01 |
| **Chronic disease status** |  |  |  |  |  |
| No chronic disease (ref) |  |  |  |  |  |
| One or more chronic disease(s) | 0.039 | 0.004 | 0.002 | 0.004 | 0.019 |
| **Healthcare access** |  |  |  |  |  |
| Insufficient (ref) |  |  |  |  |  |
| Sufficient | -0.029 | 0.005 | 0.018 | -0.02 | -0.002 |
| **Gender** |  |  |  |  |  |
| Male (ref) |  |  |  |  |  |
| Female | 0.003 | 0.004 | 0.828 | -0.006 | 0.008 |

F = 2.779, p=0.007 R^2^ = 0.002

Table S17. Full model including non-significant predictors for EQ VAS change (T2-T1).

|  |  |  |  | 95% Confidence Interval | |
| --- | --- | --- | --- | --- | --- |
|  | Beta | Std. Error | Sig. | Lower Bound | Upper Bound |
| (Constant) |  | 0.558 | 0.018 | -2.414 | -0.226 |
| **Age category** |  |  |  |  |  |
| 18-34 | 0.015 | 0.542 | 0.275 | -0.47 | 1.654 |
| 35-54 | 0 | 0.388 | 0.971 | -0.776 | 0.747 |
| 55-75 (ref) |  |  |  |  |  |
| **SES** |  |  |  |  |  |
| High (ref) |  |  |  |  |  |
| Low | -0.006 | 0.601 | 0.664 | -1.439 | 0.917 |
| Middle | 0.016 | 0.382 | 0.217 | -0.278 | 1.22 |
| **Chronic disease status** |  |  |  |  |  |
| No chronic disease (ref) |  |  |  |  |  |
| One or more chronic disease(s) | 0.017 | 0.36 | 0.161 | -0.201 | 1.211 |
| **Healthcare access** |  |  |  |  |  |
| Insufficient (ref) |  |  |  |  |  |
| Sufficient | -0.005 | 0.445 | 0.684 | -1.053 | 0.691 |
| **Gender** |  |  |  |  |  |
| Male (ref) |  |  |  |  |  |
| Female | -0.025 | 0.355 | 0.042 | -1.416 | -0.025 |

F = 1.333, p=0.230 R^2^ = 0.000
